# Outcomes and Device Usage for Fully Automated Internet Interventions Designed for a Smartphone or Personal Computer: The MobileQuit Smoking Cessation Randomized Controlled Trial

**DOI:** 10.2196/13290

**Published:** 2019-06-06

**Authors:** Brian G Danaher, Milagra S Tyler, Ryann C Crowley, Håvar Brendryen, John R Seeley

**Affiliations:** 1 Prevention Science Institute University of Oregon Eugene, OR United States; 2 Oregon Research Institute Eugene, OR United States; 3 Center for Digital Mental Health University of Oregon Eugene, OR United States; 4 Norwegian Centre for Addiction Research University of Oslo Oslo Norway

**Keywords:** tobacco, smoking, internet, eHealth, mHealth, smartphone, device

## Abstract

**Background:**

Many best practice smoking cessation programs use fully automated internet interventions designed for nonmobile personal computers (desktop computers, laptops, and tablets). A relatively small number of smoking cessation interventions have been designed specifically for mobile devices such as smartphones.

**Objective:**

This study examined the efficacy and usage patterns of two internet-based best practices smoking cessation interventions.

**Methods:**

Overall, 1271 smokers who wanted to quit were randomly assigned to (1) MobileQuit (designed for—and constrained its use to—mobile devices, included text messaging, and embodied tunnel information architecture) or (2) QuitOnline (designed for nonmobile desktop or tablet computers, did not include text messages, and used a flexible hybrid matrix-hierarchical information architecture). Primary outcomes included self-reported 7-day point-prevalence smoking abstinence at 3- and 6-month follow-up assessments. Program visits were unobtrusively assessed (frequency, duration, and device used for access).

**Results:**

Significantly more MobileQuit participants than QuitOnline participants reported quitting smoking. Abstinence rates using intention-to-treat analysis were 20.7% (131/633) vs 11.4% (73/638) at 3 months, 24.6% (156/633) vs 19.3% (123/638) at 6 months, and 15.8% (100/633) vs 8.8% (56/638) for both 3 and 6 months. Using Complete Cases, MobileQuit’s advantage was significant at 3 months (45.6% [131/287] vs 28.4% [73/257]) and the combined 3 and 6 months (40.5% [100/247] vs 25.9% [56/216]) but not at 6 months (43.5% [156/359] vs 34.4% [123/329]). Participants in both conditions reported their program was usable and helpful. MobileQuit participants visited their program 5 times more frequently than did QuitOnline participants. Consistent with the MobileQuit’s built-in constraint, 89.46% (8820/9859) of its visits were made on an intended mobile device, whereas 47.72% (691/1448) of visits to QuitOnline used an intended nonmobile device. Among MobileQuit participants, 76.0% (459/604) used only an intended mobile device, 23.0% (139/604) used both mobile and nonmobile devices, and 0.1% (6/604) used only a nonmobile device. Among QuitOnline participants, 31.3% (137/438) used only the intended nonmobile devices, 16.7% (73/438) used both mobile and nonmobile devices, and 52.1% (228/438) used only mobile devices (primarily smartphones).

**Conclusions:**

This study provides evidence for optimizing intervention design for smartphones over a usual care internet approach in which interventions are designed primarily for use on nonmobile devices such as desktop computers, laptops. or tablets. We propose that future internet interventions should be designed for use on all of the devices (multiple screens) that users prefer. We forecast that the approach of designing internet interventions for mobile vs nonmobile devices will be replaced by internet interventions that use a single Web app designed to be responsive (adapt to different screen sizes and operating systems), share user data across devices, embody a pervasive information architecture, and complemented by text message notifications.

**Trial Registration:**

ClinicalTrials.gov NCT01952236; https://clinicaltrials.gov/ct2/show/NCT01952236 (Archived by WebCite at http://www.webcitation.org/6zdSxqbf8)

## Introduction

### Background

Many current best practice smoking cessation programs use fully automated internet interventions designed for personal computers (nonmobile devices such as desktop computers, laptops, and tablets) that provide media-rich, multifaceted content [1-7]. Owing to their substantial reach via the internet, these interventions offer the promise of helping large number of smokers who want to quit [8-11]. However, benefits derived from these internet interventions are probably reduced because they are delivered largely for personal computers that are not readily accessible during a user’s everyday routine. Moreover, the interventions typically expect users to take the initiative to access the program. In contrast, just-in-time mobile internet interventions allow users to take the intervention with them during their everyday routines [12,13]. Mobile interventions take the initiative to proactively send or *push* content to users, including program reminders, strategy refreshers, and encouraging text messages [1,2,10,14-20]. Although mobile health interventions introduce new opportunities, they also come with some limitations. For example, the relatively smaller screens may require adaptations from traditional Web content in terms of shorter text and simpler graphics. A relatively small number of smoking cessation interventions reported in the research literature have been designed specifically for mobile devices [16], and to our knowledge, there are no direct comparisons of interventions designed for smartphones (mobile devices) vs interventions designed for desktop computers (nonmobile devices) previously reported. Finally, the context for this discussion is that most US adults own multiple *information devices*: (almost 77% use a smartphone, 75% use a desktop or laptop computer, and almost 50% use tablets [21]), and they use these multiple devices sequentially as well as at the same time [22].

### Aims of This Research

This study examined the efficacy and usage patterns (including devices used to visit) of 2 internet interventions for smoking cessation both of which used best practice tobacco cessation content. The MobileQuit intervention was optimized for smartphones, whereas the QuitOnline intervention represented a usual care internet intervention in that it was designed primarily for use on nonmobile PCs (desktop, laptop, or tablet computers).

## Methods

### Participants Recruitment/Enrollment

A nationwide internet-based marketing campaign used Google AdWords, Reddit, Smokefree.gov, and ORI.org. Respondents completed an internet-based registration procedure (screening survey, steps validating a functional email account and a cellphone number, informed consent, contact information, and baseline assessment) before being assigned to condition via computer-generated randomization (not personal preference; see Multimedia Appendix 1). The study protocol was approved by the ORI Human Subjects Institutional Review Board (Assurance Identification #FWA00005934).

The eligibility criteria were as follows: (1) aged ≥18 years, (2) cigarettes were the primary tobacco product, (3) smoked ≥5 cigarettes/day for the previous 6 months, (4) smoked in the last 7 days, (5) wanted to quit smoking in next 14 days, (6) active users of a smartphone (iPhone or Android) and a personal computer or tablet, (7) willing to receive up to 150 text messages over 6 months of the program, (8) able to access the internet, (9) not have another household member participating in the research project, (10) have a valid personal email address, (11) have a valid mobile phone, and (12) US resident.

#### Tailored Welcome Messaging

Each participant received a welcome message announcing their treatment program assignment (Textbox 1). This message was tailored (with emphasis added) based on the treatment assignment and the type of device the participant used during screening.

**Tailored welcome messages.**

If randomized to QuitOnline and the device being used at screening is a smartphone:

- *Congratulations you have been assigned to the stop smoking program designed especially for you to use on your desktop or tablet. We have sent you an email to confirm your participation and to help you get to your program from your desktop.*

If randomized to QuitOnline and the device at screening is not a smartphone:

- *Congratulations, you have been assigned to the QuitOnline program designed especially for you to use on your desktop or tablet. Please click on the Get Started button start using the program. We have also sent you an email to confirm your participation, and so you can get back to the program anytime you want.*

If randomized to MobileQuit and device at screening is a smartphone:

- *Congratulations, you have been assigned to the stop smoking program designed especially for you to use on your smartphone. Please click on the Get Started button to start using the program. We have also sent you an email to confirm your participation, and so you can get back to the program anytime you want.*

If randomized to MobileQuit and device at screening is not a smartphone:

- *Congratulations, you have been assigned to the stop smoking program designed especially for you to use on your smartphone. We have sent you an email to confirm your participation, and so you can get to your program from your smartphone.*

### Intervention Conditions

The 2 internet interventions presented very similar best practice smoking cessation content based on cognitive behavior therapy features (see Table 1) including many of the same interactive and multimedia features (Table 2). Both emphasized the phases of quitting—Preparing to Quit, Quitting, Maintaining Abstinence, and Retooling if lapse.

**Table 1:** Cognitive behavior therapy ingredients in both internet interventions.

Cognitive behavior therapy ingredients	Features	Example
Explanation of the treatment model^a^	Display text and animation and frequently asked questions (FAQs).	Overview of preparing to quit, quitting, and maintaining nonsmoking.
Goal setting^a,b,c^	Display text, assign stars to list of choices to choose which strategies to use, and narrow choice via series of questions.	Set goals to quit smoking and maintain nonsmoking.
Tracking^b^	Periodic notification messages asking user to reply and view summary charts of key ratings.	Track smoking/nonsmoking status and track temptation (high smoking urge) situations.
Pleasant activities^a,b^	Display text, identify activities using a *list activity* that permits typing description of activity or choose from prepopulated list items.	Identify and plan for situations that trigger smoking urges.
Self-defeating thoughts^b^	Display text and FAQs, view animations showing procedures to identify and interrupt downward spirals, and videos of coping models.	Identify and interrupt downward spirals that lead to smoking.
Positive thoughts^b^	Display text and FAQs and videos of coping models.	Focus on being smokefree.
Stress management^b^	Display text and FAQs and videos of coping models.	Two brief relaxation strategies.
Maintenance plan^b^	Choose strategies to use and sign commitment contract.	Personal plan to maintain nonsmoking.
Relapse plan^a,b^	Review circumstances of lapse, list what to do differently, and sign commitment contract.	Plan for smoking slips.

^a^Increasing awareness (destigmatizing/normalizing).

^b^Providing opportunities for corrective experiences.

^c^Encouraging repeated practice.

**Table 2:** Participant engagement activities in both internet interventions.

Activities	Functions	Examples
List activities	Encourage creation of personal lists.	Lists of pleasant activities, list of supporters, reasons for wanting to feel better, contributing factors, high-tension situations, and warning signs.
Expand-collapse (accordion) activities	Enable exploration of additional detail on topics of interest.	Frequently asked questions, myths and facts.
Drag and drop activity	Provide interactive experience to test discrimination.	Differences between extreme thoughts and everyday concerns.
Goal-setting activity	Interactive steps for selecting goals.	Number of pleasant activities to accomplish each day and the strategies that worked.
Practice change activities	Doing homework tasks in normal routine.	Identify a downward spiral, practice relaxation, and anticipate and savor activities.
Behavior tracking	Chart data over time to identify patterns and show progress.	Daily tracking of smoking status plotted in a chart.
Host videos	Provide *human touch* and highlight topics in each session.	Host videos at the start of each session.
Testimonial videos	Coping models overcome challenges to quit smoking using strategies from program.	Other smokers’ experiences, for example, doing more fun activities and managing mood patterns and stress.
Animated tutorials	Provide explanation for underlying models for change.	Show downward mood spiral and how it can be caught and managed at critical choice points.

#### MobileQuit Condition (Designed for Smartphone Delivery)

The MobileQuit condition used an integrated mobile Web app and text messaging intervention designed for a smartphone’s Web browser and had an appearance and functionality similar to what would be found on a native app (eg, button on desktop for launch). Web apps are relatively uncomplicated to update, they use similar designs and programming across iOS and Android operating systems, and they permit unobtrusive monitoring of program usage [23]. As we wanted to examine differences by device type, our log-in system attempted to constrain access so that only smartphones could be used to access the MobileQuit program.

##### Information Architecture

**Figure 1:**
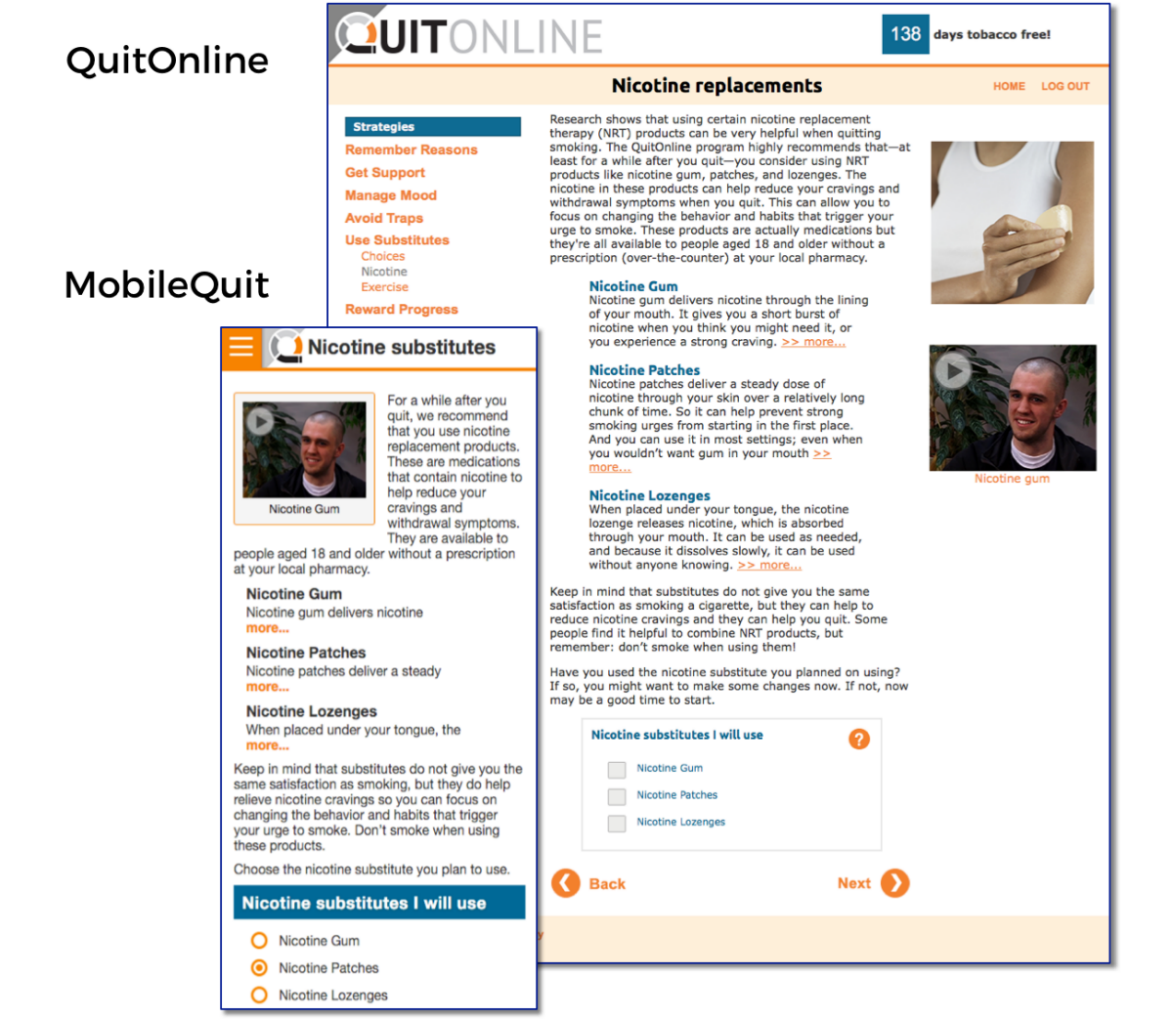
Screenshot 1 of MobileQuit and QuitOnline.

**Figure 2:**
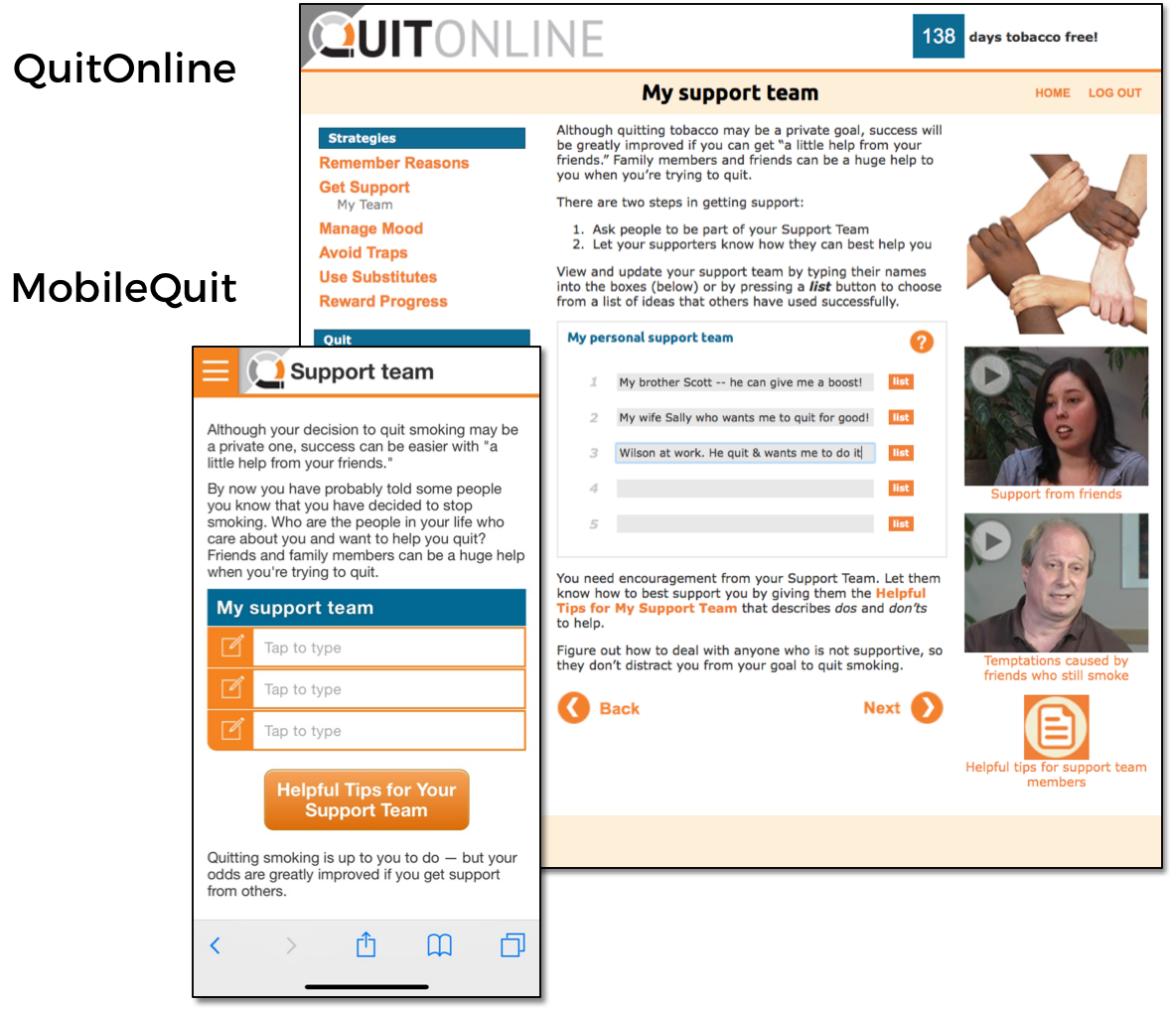
Screenshot 2 of MobileQuit and QuitOnline.

**Figure 3:**
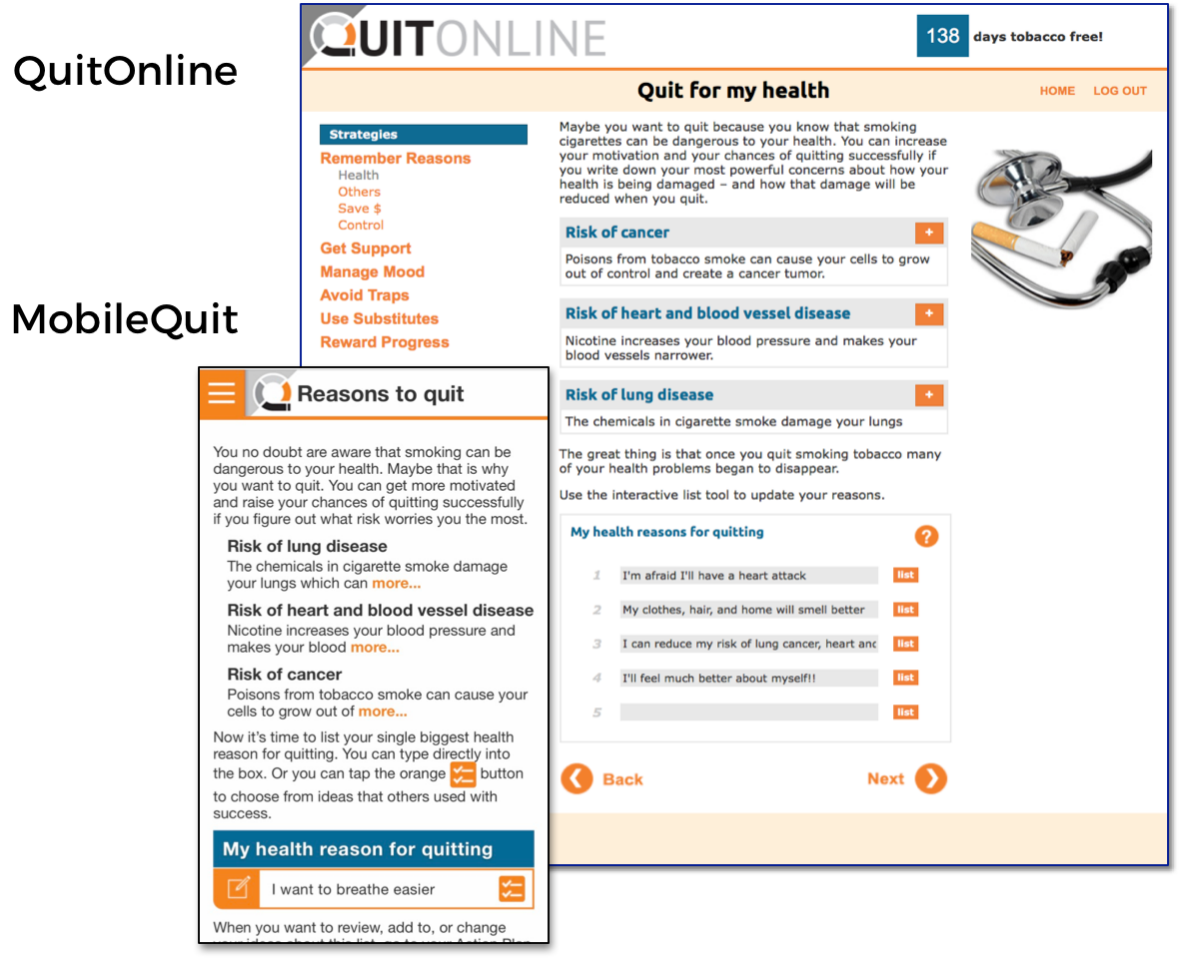
Screenshot 3 of MobileQuit and QuitOnline.

**Figure 4:**
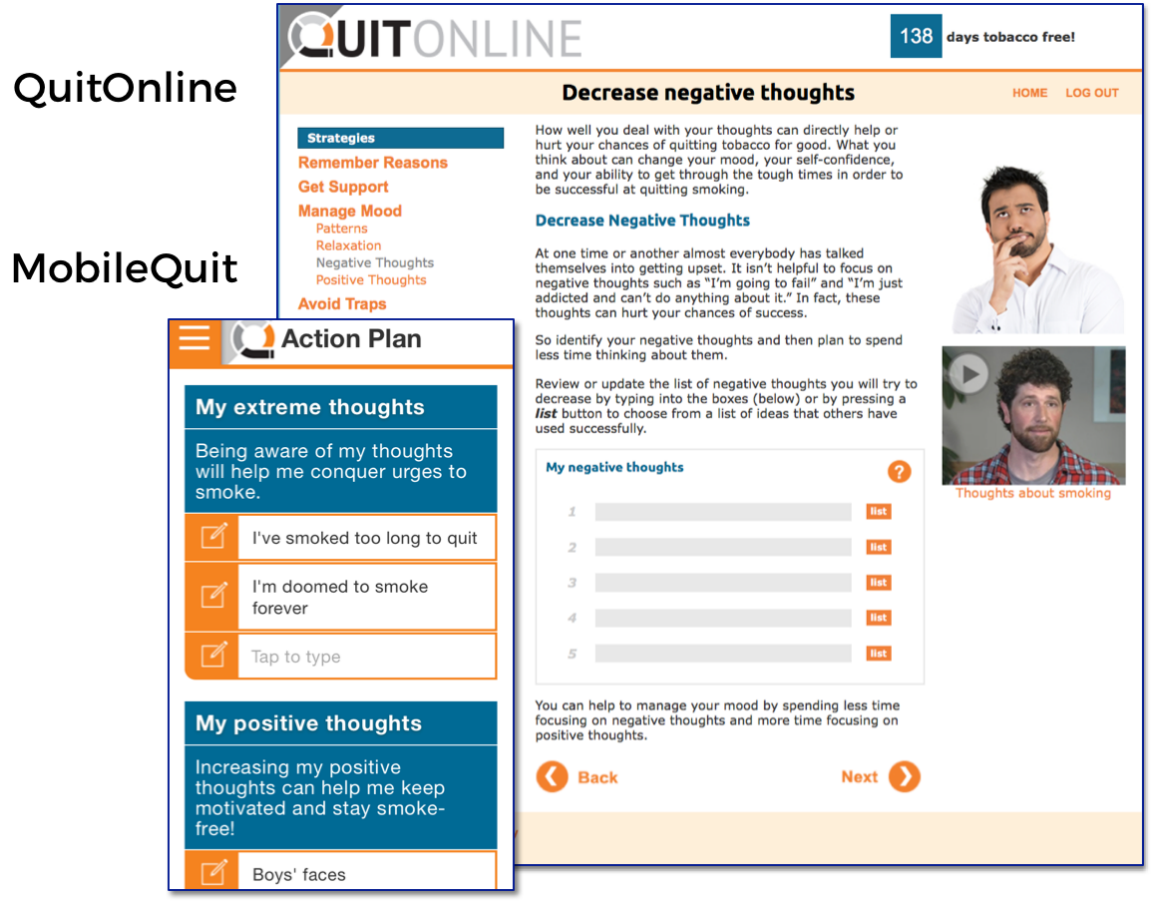
Screenshot 4 of MobileQuit and QuitOnline.

MobileQuit used a tunnel information architecture [23-25] that defined the step-by-step order in which the program was delivered over time, similar to the one used by Brendryen et al [1,2] in their efficacious *Happy Ending* smoking cessation projects. Major *Topics of the Day* could be viewed for a single day, and then excerpts were available as an ongoing reference in the program’s Library and Action Plan. Examples are displayed in Figures 1-4.

##### Lapse Management Using the Detective Activity

**Figure 5:**
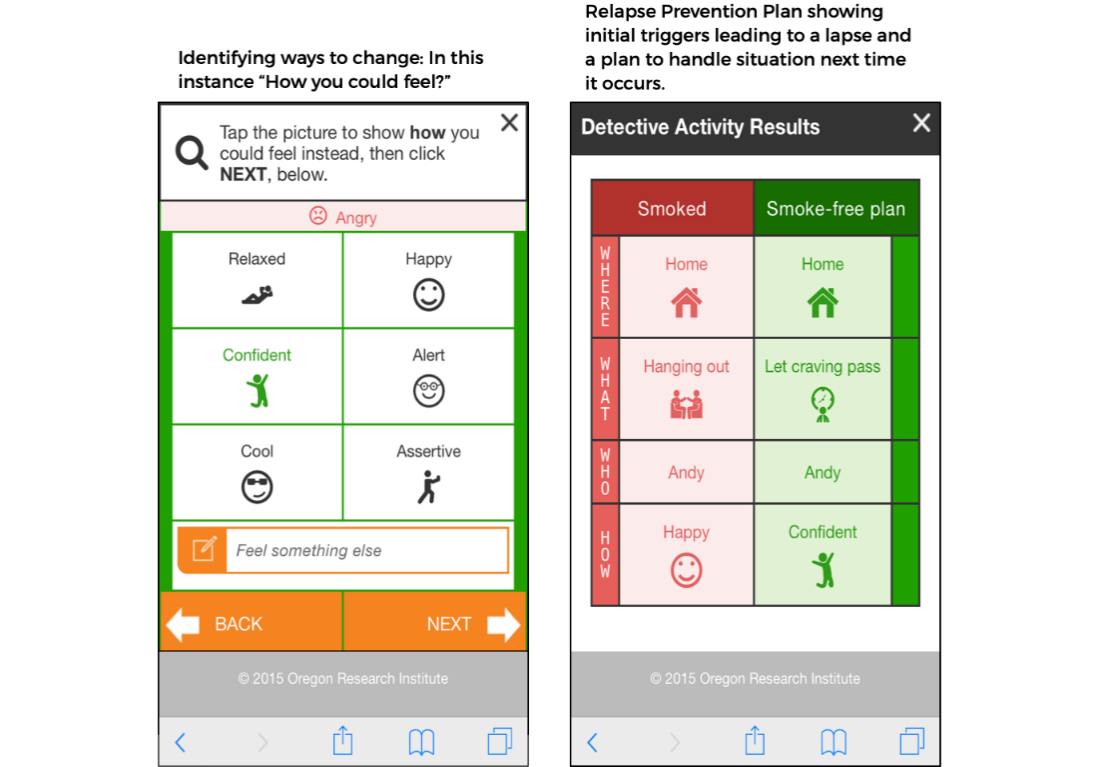
MobileQuit's detective activity.

Participants who reported experiencing a lapse were encouraged to use the program’s *Detective Activity* —an interactive wizard that asked a series of questions to help elicit the circumstances of the slip (Figure 5) to create a personal lapse-prevention plan. Participants could use the Detective Activity multiple times.

##### Text Message Content and Schedule

**Figure 6:**
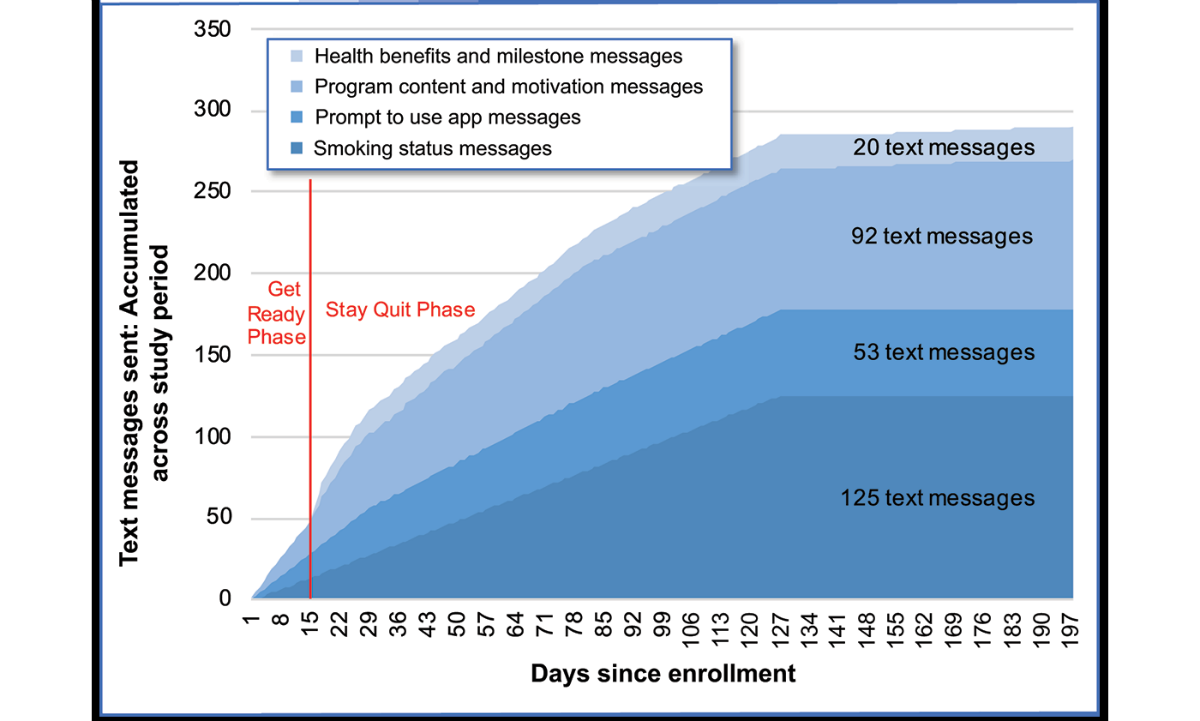
Standard regimen of 290 text messages planned to be sent to MobileQuit participants by message type.

Participants were sent text messages that synchronized with their program’s predefined tunnel schedule. As shown in Figure 6, a total of 290 text messages composed of 4 types of content were scheduled over the 6-month study period. Additional text messages were sent if the participant did not view certain program content, did not quit on the quit date, reported a lapse, reset the program’s clock, replied to smoking status texts, or was scheduled for a follow-up assessment. Participants could opt out of receiving text messages at any time without dropping out of the study.

#### QuitOnline Condition (Designed for Desktop, Laptop, and Tablet)

The QuitOnline personal computer condition was an internet intervention that used interactive and multimedia components to deliver best practice smoking cessation content. Adapted from the efficacious MyLastDip smokeless tobacco cessation program [26,27], QuitOnline used a hybrid matrix-hierarchical information architecture [24] that enabled participants to freely examine available content. Participants were sent automated email reminders to visit their program following periods of inactivity or when they set a quit date.

Although intended for use on desktop computers, QuitOnline adjusted its functionality somewhat when used on a tablet to enable touch control, entering/editing text, and playing videos. It did not automatically adjust its appearance to fit the smaller screens of mobile devices.

### Usability Testing

Both single-session and longitudinal usability testing methods were used. During single-sessions, usability testers (N=6; as recommended by Nielsen [28]) met in a research laboratory with a trained research staff member while interacting with the program and using the think-aloud procedure [29]. Consistent with use cases in usability testing [30,31] and experience sampling methods [32,33], testers followed the longitudinal usability approach that asked them to be engaged with the program during their normal routine over several weeks while keeping detailed notes. Example use cases for MobileQuit included not answering, quitting early, lapsing, and answering 2-way text messages. Testers also completed structured interviews at the end of the test period.

**Table 3:** Schedule of assessments

Assessments	Screening and Baseline	3-month assessment	6-month assessment
Socio-demographics	X^a^	—^b^	—
Past tobacco use	X	—	—
Current tobacco use	X	X	X
Quit smoking status^c^	X	X	X
Nicotine dependence	X	—	—
Self-efficacy	X	X	X
Readiness to quit	X	—	—
Depression status	X	X	X
Alcohol use	X	—	—
Cannabis use	X	—	—
Helpfulness, usability and satisfaction	—	X	—
Use of other treatments	X	X	X
Device used to access program^d^	X	X	X
Use of program content^d^	X	X	X

^a^Indicates when the assessment occurred.

^b^Not applicable.

^c^Measured via texts and return user questions from enrollment through 6-month assessment.

^d^Measured continuously and unobtrusively from enrollment through 6-month assessment.

### Assessment Plan and Measures

Baseline assessment was completed before randomization, and participants were sent an email reminder with the URL to encourage completion of follow-up assessments at 3 and 6 months (see Table 3). If a follow-up assessment was not completed after 2 weeks, research staff attempted to complete an assessment by phone. Any participant who did not complete a follow-up assessment within 45 days of its scheduled date was determined to have failed to complete that assessment. Participants received US $20 for each completed follow-up assessment and an additional US $20 if they completed both assessments. Remuneration was not tied to quitting smoking.

#### Sociodemographics

Data were collected on participant age, gender, race/ethnicity, marital status or long-term romantic relationship with a partner, and educational background.

#### Internet Usage

At both screening and baseline, the participants were asked about how they accessed the internet. For example, eligibility was determined in part by self-reported use of smartphones as well as other types of computers to access the internet. In addition, a baseline question was asked: “Overall, when you use the internet, do you do that mostly using your smartphone or mostly using some other device like a desktop, laptop or tablet computer?” Answer options included mostly on smartphone, mostly on something else, both equally, depends, or don’t know.

#### Current Tobacco Use

At screening, the respondents were asked about the number of cigarettes they smoked. Point prevalence self-reported smoking status was asked on all assessments: “In the past 7 days, have you smoked any cigarettes?” with answer options: no, not even a puff (scored 0) or yes (scored 1). If they reported that they had smoked, then they were asked “On average, how many cigarettes do you smoke in a day?” At follow-up, participants were asked “Since you enrolled in [assigned treatment program], when did you last smoke a cigarette?” with answer options less than 1 month ago, 1 month ago, 2 months ago, and 3 months ago.

#### Use of Other Tobacco Products, Quit Aids, and Nonassigned Treatments

The participants were asked about their use of any tobacco products other than cigarettes: “What type of tobacco products have you used in the past 7 days?” with answer options of E‑cigarettes, cigars, pipe, chew/snuff, other [open ended text permitted], or I do not use any other tobacco products. At all 3 assessments participants were also asked: “Are you currently using any of the following to help you quit smoking?” Answer options (check all that apply) included: nicotine replacement therapy (NRT), patches, lozenges, or gum; prescription medication, such as bupropion (brand name Zyban, Wellbutrin) or varenicline (brand name Chantix); formal treatment (telephone quitlines, therapy including group and individual, hypnosis, acupuncture, etc); and none of the above.

#### Past Tobacco Use

At baseline, the participants were asked about their tobacco history (years of use, number of quit attempts, and amount of use) as well as smoking by spouse/partner, by household members, and among their 5 best friends. They were also asked: “How many times have you made a serious attempt to quit smoking cigarettes for more than 24 hours in the last 3 months?”

#### Slip Plans

Participants were asked the extent to which they endorsed a series of statements at baseline and the 3-month assessment: “I expect that I might slip and smoke a cigarette”; “Even if I slip, I still expect to quit smoking for good”; and “If I slip, I have a plan to get back on track to being smoke free.” Each statement used the same endorsement options: strongly disagree (scored 0), disagree (scored 1), neither agree nor disagree (scored 2), agree (scored 3), and strongly agree (scored 4).

#### Nicotine Dependence

The 6-item Fagerstrom Test for Nicotine Dependence [34,35] was assessed at baseline. We separately examined the time of first smoke in the morning as this dependence item has been found to be highly predictive of subsequent abstinence [36].

#### Self-Efficacy

Participant self-confidence in quitting was assessed at each assessment by asking: “How confident are you that you will not be using tobacco a year from now” using a 5-point scale: not at all, a little, somewhat, moderately, and extremely. This item was used in our previous research [37] and was found to be a mediator of tobacco abstinence.

#### Readiness to Quit

The participants were asked at baseline to rate their confidence in quitting using the contemplation ladder [38] that has an 11-point scale with the following answer options: I have no thought about quitting smoking (scored 0), I think I need to consider quitting smoking someday (scored 2), should quit but not quite ready (scored 5), I am starting to think about how to reduce the number of cigarettes I smoke a day (scored 8), and I am taking action to quit smoking (scored 10).

#### Depressive Symptoms

Participant depressive symptomatology was assessed at baseline and at both follow-up assessments using the Patient Health Questionnaire-8 (PHQ-8) [39], which provides a validated measure of depression severity. The PHQ-8 asks: “Over the last 2 weeks, how often have you been bothered by any of the following problems?” with answer options of: not at all (scored 0), several days (scored 1), more than half the days (scored 2), and nearly every day (scored 3) [40,41]. A PHQ-8 score ≥10 has been found to have an 88% sensitivity and 88% specificity for major depression and typically represents clinically significant depression [42].

#### Alcohol Use

Alcohol use was assessed at baseline using a single item that asked, “On average during a typical week, how many drinks of alcohol do you have?” Heavy use was defined as ≥13 drinks/week for men and ≥7 drinks/week for women.

#### Cannabis Use

The 7-day point prevalence use of cannabis was assessed at baseline and the 3-month assessment using the question: “In the past 7 days, have you smoked cannabis (marijuana)?” which used dichotomous answer options of no, not even a puff (scored 0) or yes (scored 1).

#### Usability, Helpfulness, and Satisfaction

At the 3-month assessment, the participants were asked 2 questions about program usability and helpfulness:

“How easy was it for you to use the [MobileQuit; QuitOnline] program?” and “How helpful was the [MobileQuit; QuitOnline] program?” with answer options of not at all (scored 0), a little (scored 1), somewhat (scored 2), moderately (scored 3), and extremely (scored 4).

At the 3-month assessment, the participants in the MobileQuit condition were also asked questions about text messaging:

- “Did the MobileQuit text messages make it easier for you to quit?” with answer options of not at all (scored 0), a little (scored 1), somewhat (scored 2), moderately (scored 3), and extremely (scored 4);
- “How would you describe the number of text messages you received from MobileQuit?” using answer options of not enough, just the right number, too many, and no opinion.
- “Overall, what percentage of MobileQuit’s text messages did you read?”

The participants were also asked at 3 months about their satisfaction with their assigned program: “Would you recommend the [MobileQuit; QuitOnline] program to friends or family members who are interested in quitting smoking?” with answer options of yes (scored 1), no (scored 0), and not sure (scored 2).

#### Participant Engagement (Use of Assigned Treatment)

Both interventions unobtrusively tracked the overall number and duration of website visits from enrollment to the end of the 6-month follow-up assessment [37,43]. Visits were required to last at least 1 second to be counted, and there could be multiple visits/day. The date/time of each text message was logged automatically by the program, although it was not technically possible to determine whether the participant viewed or read a text message or for how long.

#### Device Used to Access the Program

The device used by each participant to make each program visit was assessed unobtrusively using the ScientiaMobile Wireless Universal Resource FiLe (WURFL) tool that analyzed the user agent string sent by the browser [44,45]. Consistent with Google’s method of categorizing mobile vs nonmobile devices [22,46], we considered mobile devices to include smartphones and feature phones whereas nonmobile devices included personal computers (desktop computers), laptops, and tablets.

### Statistical Analyses

The results were analyzed separately for the 3- and 6-month follow-up assessments as well as using a repeated point prevalence measure that combined 3- and 6-month assessments as a measure of more lasting abstinence. Logistic regression models were used to calculate the odds ratios for abstinence rate differences between intervention conditions, adjusting for significant baseline differences between conditions. Secondary analyses, assessing changes in cigarette usage (number of cigarettes per day) and quit attempts among participants who continued to use cigarettes, were analyzed using regression models with a covariate adjustment for baseline values.

The possible predictors of outcomes were assessed using a 2-step procedure. First, a univariate binary logistic regression was used to test the baseline participant characteristics as predictors of smoking abstinence using the repeated point abstinence at 3 and 6 months. Next, the significant predictors were tested using multivariate binary logistic regression with backward elimination. To identify any differential effects of intervention on outcomes, the multivariate test included treatment condition as well as the interaction of condition with sample characteristics.

IBM SPSS (version 24) was used for all statistical analyses, unless otherwise noted. Analyses used both intention-to-treat (ITT; in which participants who did not complete their assessments were considered to be using tobacco [47]) and Complete Cases (limited to participants who completed assessments). For the ITT analysis, there was sufficient power (.80) to detect a smoking abstinence rate difference of ≥7% between intervention conditions with alpha set to .017 (.05/3) to adjust for the 3 primary outcomes (3-month, 6-month, and repeated 3- and 6-month point prevalence rates).

## Results

### Participant Enrollment

The sample of 1271 study participants was enrolled from December 2015 to January 2017. The monthly enrollment varied considerably over time, with peak months occurring in the summer and monthly enrollment descriptive statistics as follows: mean 104.8, SD 59.9, median 83, minimum 1, and maximum 179.

### Participant Baseline Characteristics

The participant characteristics at baseline are described in Table 4. Consistent with the pattern reported in other studies of internet smoking cessation interventions [10,11], our sample was predominantly female (78%, 991/1271) and aged approximately 45 years. Most participants were married or had a long-term partner (68.3%, 867/1271), had made a quit attempt in last 12 months (75.77%, 963/1271), and had at least a high school degree (72.15%, 917/1271). The only significant between-condition difference in baseline participant characteristics was the larger proportion of participants in the QuitOnline condition who reported having a household member who smoked (37.4%, 235/638) than the MobileQuit condition (31.1%, 195/633).

### Participant Internet Usage

The screening procedure validated that all participants had functional smartphone and email service and they actively used both a smartphone and a desktop computer or tablet. A baseline question asked how participants accessed the internet. The results indicated that 56.57% (719/1271) mostly used a smartphone, 13.14% (167/1271) mostly used some other device, 22.42% (285/1271) used both a smartphone and other device equally, 7.71% (98/1271) indicated that it depends, and 0.16% (2/1271) did not know. No between-condition differences were found on these measures.

**Table 4:** Participant characteristics at screening/baseline by condition.

Participant characteristic^a^		QuitOnline (n=638)	MobileQuit (n=633)	Total (n=1271)
Age (years), mean (SD)		45.6 (12.3)	44.2 (12.9)	44.9 (12.7)
Female, n (%)		500 (78.5)	491 (77.6)	991 (78.0)
Married or have a long-term partner, n (%)		432 (67.7)	435 (68.7)	867 (68.3)
**Race/ethnicity, n (%)**				
	White, non-Hispanic	485 (76.3)	485 (76.9)	970 (76.6)
	Other	151 (23.7)	146 (23.1)	297 (23.4)
**Education, n (%)**				
	Not high school graduate	186 (29.2)	168 (26.5)	354 (27.9)
	High school graduate/some college	320 (50.2)	337 (53.2)	657 (51.7)
	Associate or bachelor’s degree	126 (19.7)	125 (19.7)	251 (19.7)
	Master’s or doctorate degree	6 (0.9)	3 (0.5)	9 (0.7)
Regularly smoked for 4 or more years, n (%)		609 (95.5)	591 (93.4)	1200 (94.4)
Cigarettes/day (previous 6 months), mean (SD)		17.9 (9.9)	17.1 (7.9)	17.5 (8.4)
Quit attempt in last 12 months, n (%)		480 (75.2)	483 (76.3)	963 (75.8)
**Currently use other nicotine products, n (%)**				
	Electronic cigarettes	140 (21.9)	128 (20.2)	268 (21.1)
	Cigar	40 (6.3)	45 (7.1)	85 (6.7)
	Pipe	4 (0.6)	9 (1.4)	13 (1.0)
	Chew/snuff	11 (1.7)	14 (2.2)	25 (2.0)
	None	311 (48.7)	305 (48.2)	616 (48.5)
**Use quit aids, n (%)**				
	Nicotine replacement	100 (15.7)	99 (15.6)	199 (15.7)
	Prescription medication	41 (6.4)	47 (7.4)	88 (6.9)
	Formal treatment	19 (3.0)	9 (1.4)	28 (2.2)
	No use	484 (75.9)	493 (77.9)	977 (76.9)
Nicotine dependence (Fagerstrom Test for Nicotine Dependence-6), mean (SD)		5.4 (2.2)	5.5 (2.2)	5.5 (2.2)
Self-efficacy/confidence, mean (SD)		2.5 (1.2)	2.6 (1.1)	2.6 (1.2)
Readiness to quit, mean (SD)		8.8 (1.6)	8.9 (1.6)	8.9 (1.6)
Depression status (Patient Health Questionnaire-8), mean (SD)		9.1 (6.3)	9.2 (6.0)	9.2 (6.1)
Heavy alcohol use, n (%)^b^		58 (9.1)	65 (10.3)	123 (9.7)
Cannabis use in last 7 days, n (%)		98 (15.4)	96 (15.2)	194 (15.3)
Spouse/partner currently smokes, n (%)^c^		189 (30.3)	177 (28.1)	366 (28.8)
Household member currently smokes, n (%)		235 (37.4)	195 (31.1)	430 (34.2)
Number of 5 best friends who smoke, mean (SD)		1.9 (1.6)	1.9 (1.6)	1.9 (1.6)

^a^Participants could refuse to answer any question.

^b^Defined as greater than or equal to 13 drinks/week for men and greater than or equal to 7 drinks/week for women.

^c^Denominator is full sample, participants without a spouse or with spouses who do not smoke=0 and participants with a spouse who smokes=1.

### Participant Flow Through the Study

**Figure 7:**
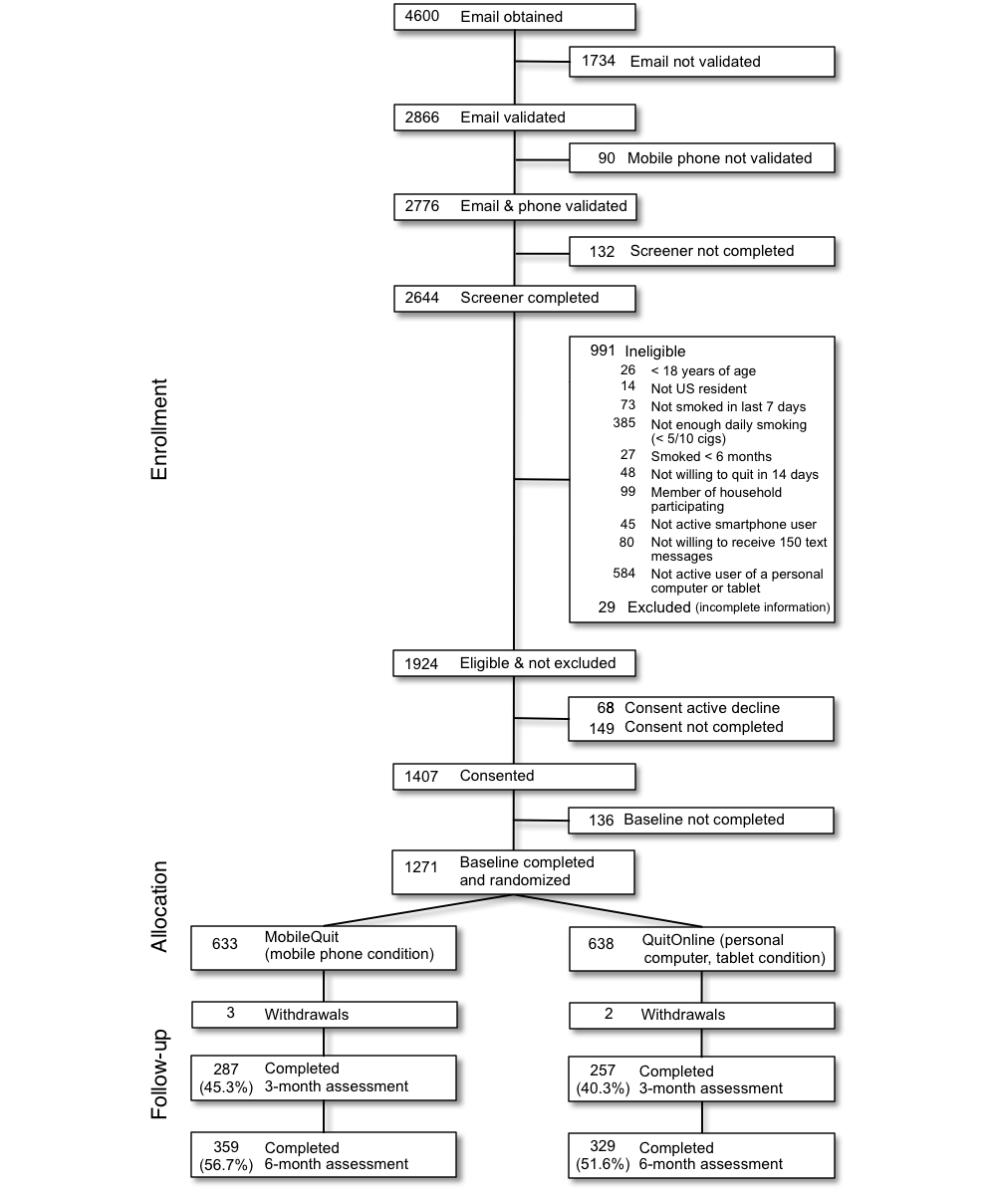
Consolidated Standards of Reporting Trials diagram depicting flow of participants through the study.

As shown in the Consolidated Standards of Reporting Trials [48] diagram (Figure 7), of the 1271 study participants initially enrolled, 42.80% (544/1271) completed the 3-month follow-up assessment, 54.13% (688/1271) completed the 6-month follow-up assessment, and 36.43% (463/1271) of participants across conditions completed both assessments.

Analysis of baseline characteristics of participants who completed assessments (Complete Cases) failed to reveal any significant differences between conditions. However, the 3-month follow-up assessment was more likely to be completed by participants who were female (46.1% [457/991] compared with 30.8% [86/279]), who reported that they did not have a long-term partner (50.6% [204/403] compared with 39.2% [340/867]), and who reported using a nicotine replacement aid (50.8% [101/199] compared with 41.32% [443/1072]). The 6-month follow-up assessment was more likely to be completed by participants who were older (mean 45.7 years, SD 12.5 compared with mean 43.9 years, SD 12.8), less depressed (PHQ-8 score: mean 8.6, SD 5.8 compared with mean 9.9, SD 6.5), had at least a college degree (61.5% [160/260] compared with 52.2% [528/1011]), used a nicotine replacement aid (61.3% [122/199] compared with 52.8% [566/1072]), and did not have a long-term partner (60% [242/403] compared with 51.4% [446/867])—especially a partner who smoked (56.1% [498/887] compared with 49.7% [182/366]).

### Tobacco Outcomes

The ITT 7-day point prevalence smoking abstinence results across both conditions were 16.05% (204/1271) at 3 months, 21.95% (279/1271) at 6 months, and 12.27% (156/1271) considering both 3 and 6 months (see Table 5). Participants in the MobileQuit condition displayed significantly greater smoking abstinence than those in QuitOnline at 3 months (adjusted OR 2.02, 95% CI 1.48-2.76; *P*<.001), at 6 months (adjusted OR 1.38, 95% CI 1.05-1.80, *P*=.02), and using repeated point prevalence at 3 and 6 months (adjusted OR 1.97, 95% CI 1.39-2.80; *P*<.001).

Complete Case smoking abstinence results across both conditions were 37.5% (204/544) at 3 months, 40.6% (279/688) at 6 months, and 33.7% (156/463) repeated point prevalence abstinence at both 3 and 6 months. MobileQuit participants displayed significantly greater smoking abstinence at 3 months (adjusted OR 2.12, 95% CI 1.48-3.03; *P*<.001) and at both 3 and 6 months (adjusted OR 1.95, 95% CI 1.31-2.91; *P*<.001) but not at 6 months (adjusted OR 1.27, 95% CI 0.93-1.73; *P*=.13). For participants who did not achieve abstinence, changes in the number of cigarettes smoked and number of quit attempts were not detected by condition at the 3- or 6-month follow-up.

**Table 5:** Smoking abstinence at follow-up assessments by condition.

Type of analysis			3-month assessment	6-month assessment	3- and 6-month assessments
**All participants (intention-to-treat)**					
	MobileQuit (n=633), n (%)		131 (20.7)	156 (24.6)	100 (15.8)
	QuitOnline (n=638), n (%)		73 (11.4)	123 (19.3)	56 (8.8)
	**Between group difference**				
		Adjusted OR^a^ (95% CI)	2.02 (1.48-2.76)	1.38 (1.05-1.80)	1.97 (1.39-2.80)
		*P* value	*<*.001	0.02	<.001
**Participants who completed assessments (Complete Case)**					
	MobileQuit, n/N (%)		131/287 (45.6)	156/359 (43.5)	100/247 (40.5)
	QuitOnline, n/N (%)		73/257 (28.4)	123/329 (34.4)	56/216 (25.9)
	**Between group difference**				
		Adjusted OR (95% CI)	2.12 (1.48-3.03)	1.27 (0.93-1.73)	1.95 (1.31-2.91)
		*P* value	*<*.001	0.128	<.001

^a^OR: odds ratio.

### Predictors and Moderators of Tobacco Outcomes

Analyses of baseline sample characteristics as possible predictors of repeated point prevalence abstinence revealed that repeated point abstinence was more likely to be reported by those who have higher levels of self-efficacy (self-confidence; beta=.35; *P*<.001; OR 1.421, 95% CI 1.176-1.718) and less likely for those with friends who smoke (beta=−.14; *P*=.030; OR 0.868, 95% CI 0.763-0.986). Only self-efficacy was significantly associated with repeated point abstinence (beta=.33; *P*=.001; OR 1.386, 95% CI 1.145-1.677) in the multivariate model. No significant interactions between intervention condition and the predictor variables were found.

### Text Message Delivery

The participants assigned to MobileQuit were sent a considerable number of text messages (mean 278.51 texts, median 295, SD 71.90, range 6-452). Sending fewer than 200 text messages was associated with 11.1% (70/633) of participants who opted out of receiving messages or who withdrew from the study.

### Participant Engagement (Use of Assigned Treatment)

The engagement metrics for all participants are presented in Table 6. The MobileQuit participants (n=633) visited their Web app program an average of 5 times more frequently than did QuitOnline participants (n=638): *z*=−20.33; *P*<.001. Among the MobileQuit participants, 90.0% (570/633) visited multiple times, 6.0% (38/633) visited once, and 4.0% (25/633) never visited. Among the QuitOnline participants, 39.0% (249/638) visited multiple times, 32.0% (204/638) visited once, and 29.0 (185/638) never visited. A different pattern emerged regarding visit duration. Owing to the brief amount of content on MobileQuit pages, 50% of visits to that program lasted ≤25 seconds. As a result, the QuitOnline participants spent significantly more time visiting their program website (*z*=−5.44; *P*<.001).

**Table 6:** Program visit engagement by condition for all participants (N=1271).

Type of analysis		Mean (SD)	Median
**Overall number of program visits**				
	QuitOnline program visits	2.32 (4.44)	1
	MobileQuit program visits	15.92^a^ (15.79)	11
**Overall duration of program visits (min)**				
	QuitOnline program visits	21.90 (35.42)	11
	MobileQuit program visits	22.34^b^ (30.46)	11

^a^Difference in overall number of website visits between QuitOnline and MobileQuit: *P*=.001 (nonparametric Mann-Whitney *U*).

^b^Difference in overall duration of website visits between QuitOnline and MobileQuit: *P*<.001 (nonparametric Mann-Whitney *U*).

**Table 7:** Visits to Web program by device type and condition.

Type of analysis				QuitOnline visits (n=438), n (%)		MobileQuit visits (n=604), n (%)^a^
**Device used for visit**						
	**Nonmobile devices**					
		Desktop computer	*500 (34.5)* ^b^		25 (0.3)	
		Tablet	*191 (13.2)*		157 (1.6)	
		Other nonmobile	0 (0)		856 (8.7)	
	**Mobile devices**					
		Smartphone	607 (41.9)		*7888 (80.0)*	
		Feature phone	149 (10.3)		*932 (9.5)*	
		Other mobile device^c^	1 (0.1)		*1 (0)*	
	Total devices		1448 (100)		9859 (100)	
**Device recommended or not**						
	Recommended		*691 (47.7)*		*8821 (89.5)*	
	Not recommended		757 (523)		1038 (10.5)	
	Total devices		1448 (100)		9859 (100)	

^a^Among the original total of 10,081 MobileQuit visits a device could not be measured in 38 instances and another 184 very short visits were associated with a robot device. The remaining 9859 sessions described in this table represent 97.8% of the original total of MobileQuit visits and 100% of QuitOnline visits.

^b^Text formatted in italics indicate devices classified as fitting the more broadly defined recommended group of devices for each treatment condition (mobile vs nonmobile).

^c^Two visits were recorded—one for each condition—as having been made by a mobile device without any additional details. We listed these 2 episodes in order to provide as comprehensive an account as possible.

The MobileQuit participants were instructed to use a smartphone to visit their program whereas the QuitOnline participants were told to use a desktop computer or tablet. Table 7 describes the devices participants used to visit their program according to the ScientiaMobile WURFL validation tool [44,45], grouped as mobile or nonmobile. Consistent with the MobileQuit’s built-in constraint, 89.45% (8820/9859) of the MobileQuit visits were made using the intended mobile device (80% [7888/9859] used a smartphone and 9.45% [932/9859] used a feature phone) whereas 47.7% (691/1448) of QuitOnline visits used the intended nonmobile device (*χ*_1_^2^=1645.9; *P*<.001). Analyses of within-participant usage patterns revealed that among the MobileQuit participants, 76.0% (459/604) used only an intended mobile device (primarily a smartphone) across all visits, 23% (139/604) used both mobile and nonmobile devices, and 0.1% (6/604) used only a nonmobile device. Among the QuitOnline participants, 31.3% (137/438) used only an intended nonmobile device across all visits, 16.7% (73/438) used both mobile and nonmobile devices, and 52.1% (228/438) used only a mobile device (primarily a smartphone).

### Usability, Helpfulness, and Satisfaction

At 3 months, both programs were described as being easy to use: MobileQuit participants (n=283, mean 3.27, SD 1.04; Somewhat easy=13.1%, Moderately easy=23.0%, and Extremely easy=57.2%) and QuitOnline participants (n=235, mean 3.03, SD 1.26; Somewhat easy=15.3%, Moderately easy=20.4%, and Extremely easy=51.5%). Similar results were obtained for program helpfulness: MobileQuit participants (n=281, mean 2.82, SD 1.20; Somewhat helpful=19.2%, Moderately helpful=27.4%, and Extremely helpful=37.7%); QuitOnline participants (n=234, mean 2.62, SD 1.36; Somewhat helpful=20.1%, Moderately helpful=23.9%, and Extremely helpful=35.5%).

The MobileQuit participants reported that they read 84.5% of the text messages they received (n=278, SD 24.6), that they were satisfied with the number of texts received (n=281, mean 2.46, SD 1.36; Not enough=5%, Just the right number=56.6%, Too many=33.1%, and No opinion=5.0%), and that receiving text messages made it easier for them to quit smoking (n=281, mean 2.46, SD 1.36; Somewhat=18.9%, Moderately=26.0%, and Extremely=29.2%). Significantly more MobileQuit participants (n=286; Yes=87%, No=5%, and Not sure=7%) reported they would recommend their program to “friends or family members who are interested in quitting smoking” than QuitOnline participants (n=253; Yes=80%, No=6%, and Not sure=14%): *χ*^2^=6.6, *P*=.036.

### Use of Other Tobacco Products, Quit Aids, and Nonassigned Treatments

The participants in the 2 conditions reported very similar patterns of using other tobacco products, quit aids, and nonassigned quit smoking treatments. The most frequently listed other tobacco products at the 3- and 6-month assessments were *Ecigs* and Other. Among the MobileQuit participants, 15% reported using *Ecigs* at 3 months and 13% at 6 months; 16% reported using *Other* at 3 months and 12% at 6 months. Among the QuitOnline participants, 16% reported using *Ecigs* at 3 months and 12% at 6 months; 19% reported using *Other* at 3 months and 19% at 6 months.

The most frequently listed quit aid at the 3- and 6-month assessments was NRT. Among MobileQuit participants, NRT use was reported by 27% of the participants at 3 months and 19% at 6 months. Prescription use was reported by 9% of the participants at 3 months and 6% at 6 months, and Formal quit smoking treatment use was reported by 4% of the participants at 3 months and 3% at 6 months. Among the QuitOnline participants, NRT use was reported by 21% at 3 months and 17% at 6 months. Prescription use was reported by 10% of the participants at 3 months and 6% at 6 months, and Formal quit smoking treatment use was reported by 5% at 3 months and 3% at 6 months.

## Discussion

### Principal Findings

Overall, the smoking cessation rates and absolute smoking abstinence levels for the 2 well-matched (fully automated, best practice content) smoking cessation programs were consistent with results reported in meta-analyses of other internet smoking cessation interventions [10,11,16,49]. However, the MobileQuit intervention for mobile devices was significantly more effective in encouraging smoking cessation than the QuitOnline designed for use on devices other than mobile devices. Specifically, ITT results of the MobileQuit participants displayed significantly greater smoking cessation than the QuitOnline participants at all follow-up assessments: 20.7 vs 11.4% at 3 months, 24.6% vs 19.3% at 6 months, and 15.8% vs 8.8% at 3 and 6 months. Similarly, Complete Case results significantly favored MobileQuit at both 3 months (45.6% vs 28.4%) and the combined 3- and 6-month assessments (40.5% vs 25.9%). However, the advantage for MobileQuit (43.5% vs 34.4%) at 6 months did not reach significance.

The participants in each condition found their treatment program acceptable, both in terms of helpfulness (MobileQuit=84.3%; QuitOnline=79.5%) and ease of use (MobileQuit=87.2%; QuitOnline=93.3%). The small between-condition differences in helpfulness and ease of use did not reach significance. Significantly more participants in MobileQuit than in QuitOnline (87.4% vs 79.8%) reported they would recommend the program to their friends/family interested in quitting.

There were 2 striking differences in usage pattern between the 2 intervention groups. First, not surprisingly, because the intervention included a built-in validation tool designed to try to constrain its use to smartphones, almost all MobileQuit visits occurred using that recommended device. In marked contrast, visits to the QuitOnline program—which did not constrain device type—showed considerable variability in being accessed using recommended as well as nonrecommended devices (including smartphones and other mobile devices).

Second, the MobileQuit participants visited their program website an average of 5 times more often than the QuitOnline participants. Stated differently, the MobileQuit intervention was used more frequently but in smaller doses/shorter visits compared with the QuitOnline intervention.

### Limitations

There are several limitations of this study that are worth noting. First, self-reported smoking abstinence was not validated by biochemical measures. However, most published tobacco cessation programs rely on self-reported data, and Glasgow et al [50] as well as the Society for Research on Nicotine and Tobacco (SRNT) Subcommittee on Biochemical Verification [51] has recommended that biochemical validation need not be required when a study’s self-help design makes it impractical, when demand characteristics are not likely to differentially affect reports by condition, or when accurate estimates of tobacco use can be obtained using multiple self-reported measures.

The participants in this study were an average age of 45 years and 78% were female. Although this profile is similar to participant characteristics reported in a number of other internet-based smoking cessation studies [5,10,52,53], our observed results may not generalize to younger smokers. As participants in this study agreed to be assigned to either of the internet-based smoking cessation interventions, it is also possible that our study results may not generalize to smokers who would have preferred to use only a smartphone-delivered intervention or, alternatively, only an internet intervention that did not use a smartphone. The study design that yoked users to particular types of computer devices (eg, smartphones for MobileQuit participants) may have been less convenient and possibly resulted in lower engagement and efficacy, compared with using responsive design technology that would have enabled either of the interventions to be used interchangeably on any internet-accessible computer device (desktop, laptop, tablet, and smartphone) [23].

There was also substantial assessment attrition at follow-up which, although consistent with results reported by other smoking cessation studies [10], was somewhat greater than has been reported for mobile smoking cessation interventions [16]. We also observed that 29% of the QuitOnline participants never visited their assigned program (an extreme case of nonusage attrition, [54]) compared with only 4% of the MobileQuit participants who did not visit. It is also helpful to consider these findings from the perspective of other published results. For example, QuitOnline’s 29% nonvisit rate is similar to the 20% to 25% nonvisit rate results reported by Cobb and Graham [55] for 2005 participants in a smoking cessation randomized controlled trial (RCT). This difference is also consistent with our expectation that the push or proactive outreach of MobileQuit’s text messages would encourage relatively more participant engagement. Moreover, by providing time-limited access to its Major Topics content, the MobileQuit program may have increased its perceived value of the program (the scarcity principle) to its users. The program’s tunnel design also paced program content so that it was better synchronized with the phases of quitting, which may have encouraged involvement and sustained interest.

### Strengths

This trial is one of the relatively few large-scale (N=1271) RCTs examining the efficacy of smartphone-delivered smoking cessation intervention. The interventions were designed to reliably and unobtrusively track the device used to access the program as well as the frequency and duration of each participant’s contact. Thus, the trial represents a rare instance of both describing the devices that participants used to access the internet intervention and comparing the usage pattern across device type. The use of a browser-based Web app for MobileQuit enabled us to avoid having to create 2 altogether different native apps (one for iOS devices [iPhones and iPads] and another for Android devices [smartphones and tablets]), to avoid the review and delay associated with distributing native apps via official company app stores [23], and to set the stage for a responsive design that would be usable across all devices and their operating systems. These benefits combined with the emergence of the more sophisticated *progressive Web app* [56] are encouraging more widespread use of the Web app design approach. As text messages are increasingly being delivered on nonmobile devices, a Web app plus text messaging intervention can be delivered across all devices.

### Future Directions

Although the absolute proportion of smokers who quit in this study was encouraging, additional research is warranted that examines how to encourage even more widespread smoking abstinence. This design did not permit a direct analysis of the individual and combined features of the 2 conditions. For example, impact of device type and use of text messaging on long-term smoking abstinence could be examined using a completely crossed 2×2 design (smartphone vs not smartphone and app + text messaging vs app only). It would also be helpful to examine the likely contributions of other factors that were not examined directly in our RCT (eg, tunnel vs hybrid matrix-hierarchical information architecture). In all instances, research should assess the devices and the usage patterns that participants follow to access their internet interventions.

### Conclusions

Despite the fact that this study did not pinpoint the exact design feature(s) that explain the increased efficacy of MobileQuit over QuitOnline, the study nonetheless provides evidence for the benefit of optimizing an intervention design for smartphones and other mobile devices over a usual care internet intervention designed primarily for use on nonmobile devices such as desktops, laptops, or tablets. Our study also helps to underscore that participants will use multiple devices (what Google describes as *sequential screening* [22]) irrespective of recommendations to do otherwise. As a result, we assert that future internet interventions should be designed for use on all of the devices that users prefer to access the internet. Essentially, in the increasingly multiscreen world, the approach of designing internet interventions for mobile vs nonmobile devices will be replaced by responsive-designed programs that share user data across devices and embody pervasive information architecture [22,23,57,58].

## Appendix

Multimedia Appendix 1 Multistep enrollment validation sequence for the MobileQuit randomized controlled trial.

Multimedia Appendix 2 CONSORT‐EHEALTH checklist (V 1.6.1).

## Abbreviations

ITT: intention-to-treat
NRT: nicotine replacement therapy
OR: odds ratio
PHQ-8: Patient Health Questionnaire-8
RCT: randomized controlled trial
WURFL: Wireless Universal Resource FiLe
