# Supplementary figures and images for: Outcomes and Device Usage for Fully Automated Internet Interventions Designed for a Smartphone or Personal Computer: The MobileQuit Smoking Cessation Randomized Controlled Trial
Source: J Med Internet Res. 2019 Jun 6;21(6):e13290. doi: 10.2196/13290 (PMC6594213; doi:10.2196/13290)

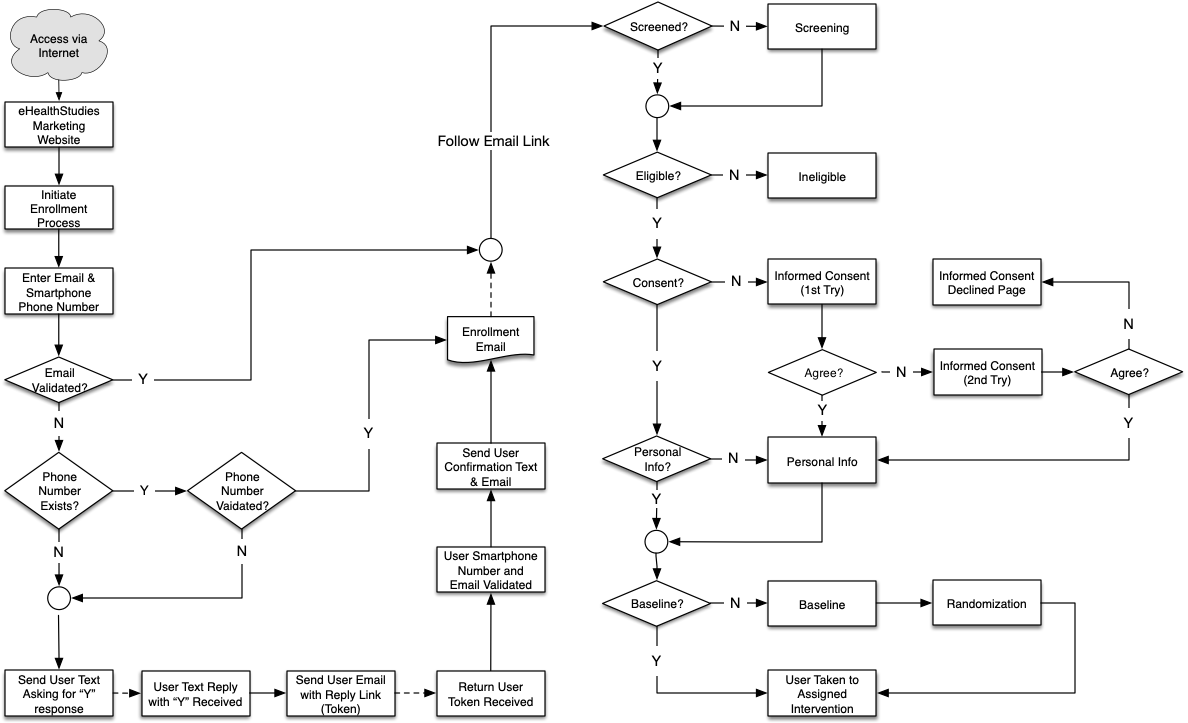

Supplement: Multimedia Appendix 1 [file jmir_v21i6e13290_app1.png]
